# A biopsychosocial approach to death, dying, and bereavement: a course on end-of-life education for medical students

**DOI:** 10.12688/mep.19140.3

**Published:** 2022-08-22

**Authors:** Simran Kripalani, Sandra Joy, Shivani Raizada, Elizabeth Cerceo

**Affiliations:** 1 Cooper Medical School of Rowan University, Camden, NJ, 18103, USA; 2 Rowan University, Glassboro, New Jersey, 08028, USA; 3 Cooper University Hospital, Camden, NJ, 08103, USA

**Keywords:** medical education, end-of-life care, death, dying, bereavement

## Abstract

**Background**: Despite the inevitable nature of death and dying, the conversations surrounding this subject are still uncomfortable for many physicians and medical students.

**Methods**: A six-week humanities-based course, “A Biopsychosocial Approach to Death, Dying, & Bereavement,” at Cooper Medical School of Rowan University, United States, which covers definitions of death and dying, the process of dying, ethical dilemmas, and new concepts of the grieving process. Through development of a curriculum using various academic and medical literature and resources, we sought to bring attention to the necessity of having a medical education curriculum on death and dying to prepare medical students for the difficult conversations and patient experiences that lie ahead of them. Qualitative data in the form of surveys and reflection papers submitted by students and quantitative data (Likert scores on course satisfaction) were collected and analyzed both pre- and post-course.

**Results**: 90.7% (49/54) of the respondents answered that they agree or strongly agree with the statement that this selective course was useful in the student’s medical education experience. The top three qualitative themes brought up the most in reflection papers (n=54) were: the utility and instruction of the course (21 times), the importance of hospice and palliative care (20 times), avoidance around topics of death (15 times).

**Conclusions** *:* Medical students are often not prepared to cope with the realities of patient loss and of caring for the patient and their families throughout the dying process. We created this course to familiarize medical students with an aspect of the medical experience that is frequently neglected in traditional medical curricula. We learned that integrating such a course can help educate medical students facilitate important conversations, teach them to act with kindness and dignity in a physician-patient setting, and enhance their personal understanding of death and dying.

## Introduction

Death is infrequently acknowledged in the field of medicine but is an experience that all medical students will encounter in some capacity as residents and practicing physicians. Despite the inevitable nature of death and dying, the conversations surrounding this subject are still uncomfortable for many physicians and medical students ^1^. Past studies have shown that many medical students believe that they are not adequately exposed to topics on death, dying, and end-of-life care ^2^. Though initiating discussions about death and appropriately supporting the patient and family is an integral part of the physicians’ careers, many students feel that it is one of the things that medical education least prepares them for ^3^. While students are taught how to reach successful medical diagnoses and are educated on the physical setting in which it is best to deliver bad news to the patient and their family, this is only a superficial, though necessary, component to patient communication. Students would benefit from greater exposure and experience in supporting patients and families with empathy ^4^. By preparing future physicians with useful skills such as learning how to cope with grief and loss, we will be able to cultivate empathy and build resilience among future physicians.

Patients prefer receiving death in a “human-oriented approach,” full of empathy and understanding, rather than the typical “organ-oriented” and “disease-oriented” diagnosis with which the medical community is more familiar ^5^. A survey of medical hematology and oncology fellowship directors reported 89% of program directors felt they themselves received nearly no formal training in the delivery of bad news and, out of the current fellows, 77% stated that they received some or nearly no training. Additionally, 63% of these program directors felt that medical schools should provide more foundational training in the subject ^6^. Providing future physicians with opportunities to receive and practice proper education and training in death and dying will help them better cope with the death of their patients and equip them with resources on how to openly talk about death, dying, and end of life care. With additional support embedded in medical education, students can prepare themselves to take care of their own emotional well-being while navigating the death of patients they directly care for ^7^. Patients who are facing the end of their life may feel more comforted by these future physicians, who will have more knowledge and education behind death and dying. This can directly lead to supporting and guiding patients dealing with uncertainty and hardship in a more sincere and humanistic manner.

Medical education plays a pivotal role in delivering these skills to future physicians. Providing student doctors with the opportunity to engage in targeted practice on strategies for handling tough conversations with patients, while maintaining professionalism, mental balance, and emotional detachment, is ideal. Our course, “A Biopsychosocial Approach to Death, Dying, & Bereavement,” was developed at Cooper Medical School of Rowan University, United States. It exposes students to thanatology and defines death from the various dimensions of human development. The course discusses definitions of death and dying, the process of dying, related ethical dilemmas, and new concepts of the grieving process. Ultimately, we identified the need for such training in the medical curriculum and created a course to help facilitate these conversations and act with kindness and dignity when doing so (see *Extended data* ^8^).

## Ethics

This study was deemed to be exempt status per the Rowan University Institutional Review Board (IRB). Because this project is for medical education and utilizes primarily qualitative data from deidentified students who enrolled in the course, Rowan IRB has determined that this project is in the exempt category. Upon enrollment of the course, students were informed that the data collected in the course would be used to enhance medical education. IRB and the Office of Medical Education deemed that no written consent would be required because the data was collected as part of normal educational activities and deidentified during analysis by OME. Much of the institution’s curriculum is novel and routine assessment of curricular elements is considered quality improvement by Rowan IRB.

## Methods

Cooper Medical School of Rowan University offers sessions to pre-clinical medical students called the Selectives in the Medical Humanities. The Selectives cover a wide array of topics from ethics and fine arts to improvisation and dance to narrative medicine and storytelling. Six to eight Selectives are offered each semester and students participate in a lottery with ranked preferences to determine enrollment. They consist of six two-hour sessions over the course of both the Fall and Spring semesters. First and second year students must select two semesters to take these humanities courses which are structured as small groups. Our course focused on expanding students’ knowledge of concepts and issues surrounding the process of death, dying, and bereavement with a goal to improve the doctor-patient relationship and personal understanding. Prior to the start of the course, students received the syllabus (see *Extended data*), which highlighted the overview, goals, objectives, expectations, and course outline. In preparation for the course, five articles were required to be read before each session (see *Extended data*). The book titled *In the Face of Death: Professionals Who Care for the Dying and the Bereaved* was used to supplement the respective sessions ^9^.

The six weekly lectures were thematically arranged. The topics covered were “The Caring Relationship,” “The Care Provider in Death Situations,” “Hospice Care,” “The Team in the Face of Death,” “Managing Sources of Stress,” and “Motivations & Rewards; Reflections on Living & Dying” ( Table 1). Small group discussion was facilitated by a lead instructor (author SJ) and focused on articles and book sections along with videos and vignettes to expressed shared experiences of the material and to solidify understanding of the topic.

**Table 1.** Course breakdown.

Week	Topic	Learning outcomes	Performance indicators of learning
1	The caring relationship	Distinguish between various models for the caring relationship (i.e., medical, biopsychosocial, holistic, palliative) Describe the impact of the relationship between society, science, and death Identify features of the Helping Relationship between the dying or grieving and the physician	Read Section I: “The Caring Relationship” found in *In the* *Face of Death: Professionals Who Care* Discuss reactions to these readings during class meeting
2	The care provider in death situations	Develop awareness of one’s own personal responses to death and be able to identify grief complications experienced by physicians	Draper, Emma, *et al.* (2019) “Relationship between Physician’s Death Anxiety and Medical Communication and Decision-Making: A systematic review,” Patient Education and Counseling, 102:266–274 Discuss reactions to these readings during class meeting
3	Hospice care	Develop awareness of Hospice Care (i.e., history of the hospice movement, nature of the work, approach to end of life care, palliative care) Know what is meant by the concept of the wounded healer; be able to identify signs of compassion fatigue, burnout, and vicarious traumatization Distinguish between the focus/treatment found in hospice versus hospital care	Read Section II: “The Care Provider in Death Situations” found in *In the Face of Death: Professionals Who Care* Brighton, Lisa Jane, *et al.* (2019) “Emotional Labour in Palliative and End-of-Life Care Communication: A Qualitative Study with Generalist Palliative Care Providers,” Patient Education and Counseling, 102: 494–502 Discuss reactions to these readings during class meeting
4	The team in the face of death	Describe ways to manage difficult patients and families, deal with pressures of work and manage impact of work stressors on staff Describe ethical issues that arise with dying patients and grieving families Identify way of coping with stressors	Ekberg, Stuart, *et al.* (2019) “Discussing Death: Making End of Life Implicit or Explicit in Paediatric Palliative Care Consultations,” Patient Education and Counseling 102: 198–206 Discuss reactions to these readings during class meeting
5	Managing sources of stress	Identify the features of optimal team functioning in death situations at a systemic level Identify various organizations that interface with the hospital and physician’s role in death situations (i.e., organ donation agency; funeral home; bereavement counseling agencies, etc.) Describe ways in which interdisciplinary teamwork can be facilitated to benefit the dying and the bereaved	Read Section III: “The Team in the Face of Death” found in *In the Face of Death: Professionals Who Care* “Patient-Centered Care: Case Studies on End of Life,” Healing Hands, Vol. 22, No.1: Winter 2018 Discuss reactions to these readings during class meeting
6	Motivations & rewards; reflections on living & dying	Describe the Initial Motivation for Working with the Dying, Bereaved Families and be able to describe the factors that make this work worthwhile Identify Diversity Issues that Arise in Work with the Dying and their Families, as well as Treatment Indications that are Culturally Sensitive Describe what constitutes “A Good Death”	Kamal, Arif, *et al.* (2016) “Prevalence and Predictors of Burnout among Hospice and Palliative Care Clinicians in the U.S.,” Journal of Pain and Symptom Management, Vol. 51 No. 4, April 2016 Discuss reactions to these readings during class meeting

In order to proceed with this study, we were reviewed for IRB approval and exempt (STUDY ID: PRO-2021-313). Students were required to complete one reflection paper at the end of the Selective (about 2-3 pages in length), reflecting on an issue that surfaced in the class discussions related to death, dying, and/or bereavement.

Assessment of the Selective is Pass-Fail and is based on attendance, engagement with the material, and participation with comments on students’ performances that can be integrated into the Medical Student Performance Evaluation (MSPE).

To understand the impact and response of the course, four semesters were studied: Fall 2019, Spring 2020, Fall 2020, and Spring 2021. Students completed an online anonymous evaluation for all courses. The survey for the Selectives was short and asked participants to evaluate the course on its usefulness in their medical education with a Likert scale. The options given on the survey included “n/a,” “strongly disagree,” “disagree,” “neutral,” “agree,” and “strongly agree.” The sample size for the Fall 2019, Spring 2020, Fall 2020, and Spring 2021 semesters were 15, 13, 14, and 15, respectively. 12/15 people responded to the end of the Selective survey question and completed reflection papers for Spring 2021. Qualitative comments regarding the course are reflected in Table 3.

**Figure 1.**
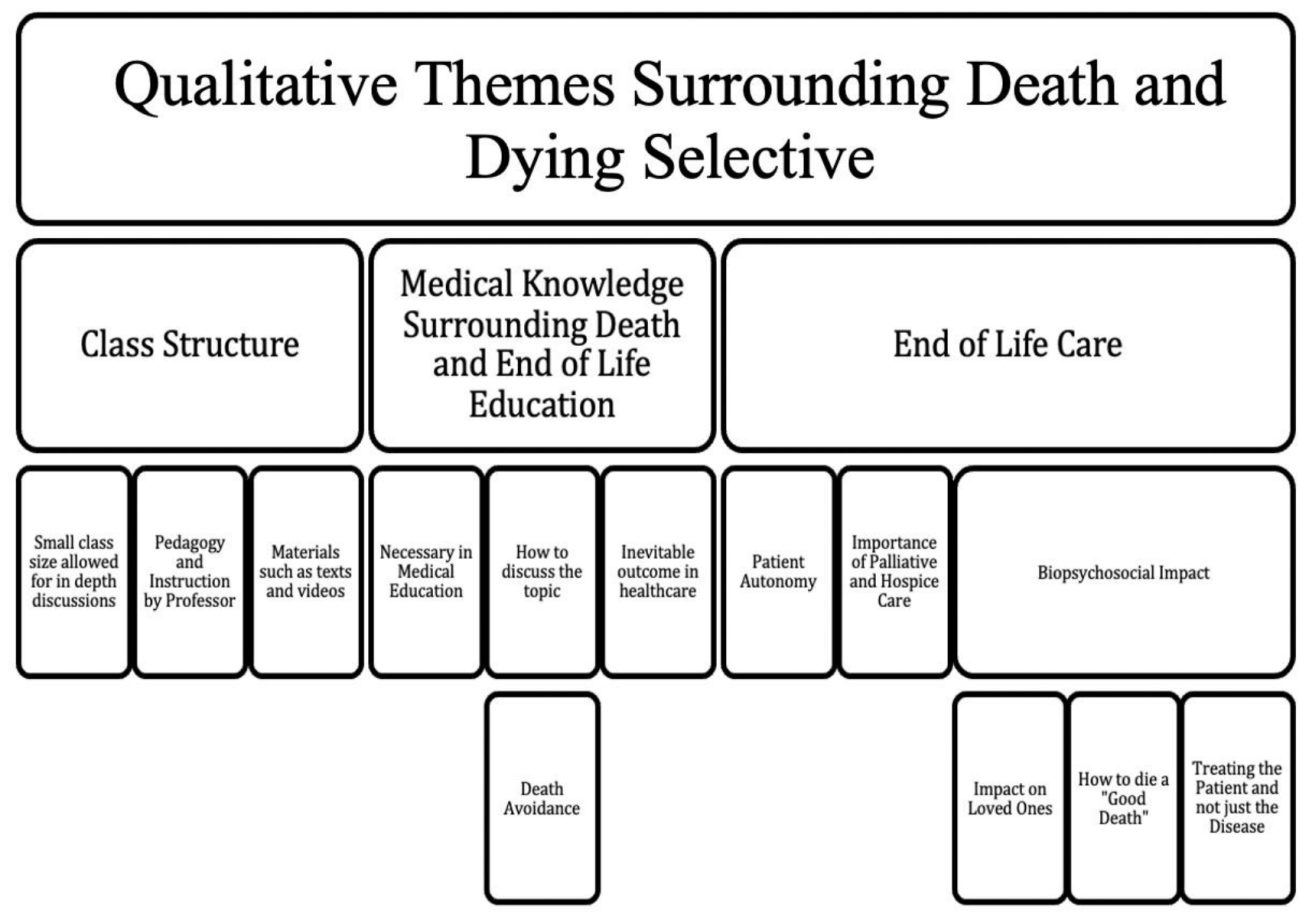
Qualitative Themes for Medical Education Selective on Death and Dying.

In addition to the survey, students were asked to complete a reflection paper detailing their experiences with conversations on death and dying prior to the class, their takeaways from the class, and how this class has impacted their mindset as a future physician. The sample size of students who submitted reflection papers for Fall 2019, Spring 2020, and Fall 2020 was 13, 15, 13, and 13, respectively. The reflection papers were then taken and analyzed for recurrent themes and commonalities. Qualitative responses from each semester were reviewed by two researchers (SK, SR) in order to identify common themes read in the papers. The two researchers developed themes *a priori*, assessed one semester of data, reviewed them together after each was conducted independently to ensure standardization in extracting themes, and then completed the coding tree with any additional themes elicited by first review. One semester was selected as a baseline as saturation of themes was achieved. A coding tree was developed and essays from subsequent semesters were assessed based on this structure of qualitative themes ( Figure 1). Examples of some comments stated by the students regarding the course are mentioned ( Table 2). Frequency of responses were tracked to pinpoint which themes were most prominent in all the semesters in this study ( Table 3)

**Table 2.** Sample of representative student comments on the course.

Sample of representative student comment on the course
It was a great learning experience on a topic that is very important and not discussed enough.
I enjoyed discussing with classmates about death, dying, and bereavement and thought this is a very important topic to learn about as almost everyone will have to encounter death at some point.
[Course] has so much insight and provided us with many unique perspectives on the topic of death and dying.
It allowed me to gain more knowledge about end-of-life care.
Very interesting, accessible
I thought the group discussions and supplemental material/material we went over and watched during class was very helpful in reinforcing any topics learned
I was challenged to really think outside of the box during this Selective. I had to consider topics about death and dying that I would not normally want to think through, and further, I had to consider these from many different perspectives.
It was great to be able to talk about our feelings about the readings and videos.

**Table 3.** Qualitative responses in end-of-semester reflection papers (n=54).

Themes	Fall 2019	Spring 2020	Fall 2020	Spring 2021	Total # of responses
Death avoidance	5	2	6	2	15
Treating a patient, not a diagnosis	5	5	2	1	13
Detached view of death	2	1	2	0	5
Importance of hospice care/palliative care	8	8	2	5	23
Holistic approach to death	2	1	3	0	6
Respecting patient autonomy	2	4	4	6	16
Quality of life	2	0	2	0	4
Impact on family	4	1	6	4	15
Provider burnout	3	2	3	6	14
What it means to die a “good death”	4	4	2	2	12
Importance of a team	2	1	3	3	9
Self-awareness	1	0	0	0	1
Psychosocial aspects of death & dying	2	0	2	2	6
Pain management	1	0	0	0	1
Compassion/empathy	3	2	2	3	10
Interpersonal connectedness	1	1	0	0	2
Comforting patients	1	1	1	2	5
Condense reading	5	0	0	0	5
Personal experiences with death	0	1	3	3	7
Learning how to grieve as a provider	3	2	3	5	13
Death of a child	2	1	2	0	5
Medical ethics	1	0	1	0	2
Afterlife	1	0	1	1	3
Unique pedagogical approach/ the utility and instruction of the course	6	7	3	7	23
Breaking bad news to patients/families	1	1	0	0	2
Changing needs of a patient	1	1	0	0	2
The impact of COVID-19 on death	0	5	1	2	8
Death anxiety	0	6	4	0	10
Necessity of this topic in med ed	0	7	1	2	10
A lack of knowledge prior to this	0	3	2	0	5
Plea for house calls	0	0	1	0	1
Could use more instances to learn from people in the field	1	0	0	1	2
Perspective	1	0	0	0	1
Humanity in medicine	1	1	0	2	4
Understanding grief	0	1	0	0	1
Course for mental preparation	0	1	0	0	1
Useful for future physicians	0	5	6	3	14
Coping mechanisms	0	2	0	0	2
Role of religion in death	0	1	0	0	1
Healing greater than curing	0	2	0	0	2
Maintaining professionalism	0	1	1	3	5
Pervasiveness of death in the medical profession	0	4	1	7	12
Mistrust in medicine from lack of access	0	0	1	0	1
Do no harm	0	0	1	0	1
Need for advance directives	0	0	1	0	1

Additional psychometrics were administered for only the Spring 2021 course. The Toronto empathy scale and Budner tolerance for ambiguity score were administered before and after the course ^10, 11^. Responses were statistically analyzed to determine if the course impacted empathy and tolerance for ambiguity.

## Results

Data was taken from four semesters (Fall 2019, Spring 2020, Fall 2020, and Spring 2021) to answer the following statement using a Likert scale: this elective was useful in the medical student’s medical education experience. Students positively evaluated the course, rating it a 4.7 out of 5 (range for other Selectives 3.8–4.9) ^8^. 90.7% (49/54) of the respondents answered that they agree or strongly agree with the statement that this Selective course was useful in the student’s medical education experience.

At the end of the course, the medical students enrolled were asked to write reflective papers focused on what they learned in the class and their observations and takeaways from the course. For the four semesters, there were 54 respondents for the survey, as well as 54 cumulative reflection papers. The top five qualitative themes that were brought up the most in the reflection papers of four semesters worth of classes (n=54 papers) were: the utility and instruction of the course (21 times), the importance of hospice and palliative care (20 times), avoidance around topics of death (15 times), respecting patient autonomy (14 times), and the impact on family and loved ones (14 times). Themes and their frequencies are shown in Table 3.

In terms of course utility and structure, students appreciated the flexible nature of the Selectives, citing that, although there was a basic schedule and syllabus during each session, there was “no rigid schedule we had to stick by which made it great because while [they] also covered the necessary curriculum, [they] managed to also tailor it to [them] at the same time. This made it all the more enjoyable and informative.” .Students also mentioned enjoying classroom discourse and one student described their experience as such: “Having a casual conversation with my peers about such an overlooked topic made me more comfortable addressing it.” Over all, comments surrounding the theme elaborated saying that the “discussion flowed easily in small groups,” that “meaningful conversations with peers were strengths,” and that the class “prepared us for a part of medicine that is often overlooked but crucial.” The differences between palliative and hospice care made an impact on the student participants. Students learned to view palliative care as, “the next step in properly caring for a patient,” rather than the “failure to provide adequate care.” On the topic of palliative care, a student wrote that “this course has helped me see that the switch to palliative care in cases such as this should not be seen as a failure to provide adequate care but rather as the next step in properly caring for a patient.”

The third common theme is regarding avoidance of topics related to death and dying. One student stated, “it is often taken for granted that medical students are comfortable with death because they chose a career path where death is inevitable,” and claimed that, “more students have death anxiety than those who do not.” They felt that there is value in, “students being able to discuss this and confront their death anxiety.” Students commonly agreed that the content of this course is applicable to them and that they are “all aware [they’ll] face death at work,” even if they do not do a fellowship surrounding palliative or hospice care.

Students understood the role physicians play in the lives of patients but also the role they play in the lives of their loved ones. Students recognized this and stated “talking to patients and their families about fatal prognosis is a key role of a physician.” Several reflection papers mentioned biopsychosocial impacts on health outcomes of the patients.

The fifth most common theme gathered from the reflection papers was of patient autonomy. Ultimately, students said that it is important for physicians to create treatment plans that allow “patients’ end of life to be in a way they can tolerate as well.” Students talked about the importance of explaining comprehensive treatment options, and while this, “may not always line up with the best treatment plan, a textbook, or statistics,” physicians must ultimately respect the patients’ autonomy.

Results of pre- and post-testing (before and after the semester elective) with the Budner Tolerance for Ambiguity scale (p=0.70) and the Toronto Empathy scale (p=0.82) were not significantly different.

## Discussion

Having conversations about dying and coping with death is something that most physicians will deal with in some capacity, yet there is a lack of medical education to prepare medical students for this important aspect of their careers ^12^. Additionally, an undercurrent in medical training often conjoins patient death with a failing on the part of the clinician rather than an expected outcome of a disease process the physician did not cause. Many students enrolled in this course emphasized the need for death education for medical students to enhance preparedness and serve as a resource for their patients. Literature shows the potential benefit in having formal coursework and bedside teaching to enhance medical student preparedness ^1, 13^. Both the quantitative and qualitative results point to a course focused on death and dying providing a supportive framework for an important issue in a future physician’s practice.

The most common theme mentioned in the reflection papers was the perceived utility and instruction of the course. In the papers, students mentioned that they appreciated how the class was conducted and its unique pedagogical approach. Some of the comments were generally reflective of structure of the Selectives such as small class size and interactive, discussion-based approach. They also gave positive course feedback on the discussions and materials used and appreciated its meaningful instruction. Discussion-based learning can increase practical knowledge, improve long term understanding, and strengthen the comfort level of applying subject materials ^6^. Students similarly reported finding value in the discussion-based classroom environment and “felt as though it provided a forum for [them] to speak openly about death and [their] fears surrounding it.” A student stated that they, “quickly became comfortable because the topic was approached with the more reasonable attitude that death is not inherently bad and that we can talk about it candidly.” The instructor (SJ) created a safe space where people felt comfortable in sharing their personal experiences and opinions on death and dying. A smaller class size allowed for a more intimate setting where students felt connected and comfortable. Another aspect of the class that students enjoyed was the content of the videos and readings for the class and the meaningful insight provided by the instructor.

 The next most common theme was the importance of hospice and palliative care in medicine. Palliative care education for medical students is crucial, and there is literature showing the importance of adequate palliative training ^1, 14, 15^. Palliative and hospice care education in medical school can lead to a more humanistic approach to clinical care and allow for learning opportunities ^16^. Students frequently referred to how the class allowed them to understand the difference between hospice and palliative care. Learning the nuances between palliative and hospice care and understanding what they both entail was valuable to several students who took the class. Students appreciated exposure to a diverse body of literature to round out discussions of hospice and bereavement care and felt they could reflect on personal experiences with dying patients and family members.

Avoidance of topics related to death was a frequently cited thematic area. Many students felt a significant level of discomfort talking about death prior to taking this class. They felt unprepared to handle this taboo topic, even though they felt that, “it is a topic that as future healthcare providers and as members of society should talk more about People who took this class were all aware of the inevitable nature of death in their profession, but felt the most unprepared to tackle this subject. Prior to taking the course they, “really had to think hard about how [they] might handle the situation or how comfortable [they] would be.” However, as the course progressed, many students felt that the lessons they have learned throughout this course “changed [their] outlook of death.” A common change in the students’ perspectives included the acceptance that of death not being the worst possible clinical outcome. By the end of the course, students felt increased confidence in handling and coping with death, dying, and bereavement. Medical students often felt unprepared to deal with their emotions and cope with patient death while maintaining professionalism. Encouraging medical schools to include topics of spirituality, religion, hospice, and palliative care can also enhance the ability for students to learn how to process their emotions in the future ^14^. “Having a casual conversation with [their] peers” made it comfortable to talk about the overlooked topic of death. Despite starting the class with death anxiety, students feel comfortable and are willing to, “discuss these topics in the future with other colleagues.”

Caring for family members and friends is a critical role as the physician must care for more than just the patient in front of them. The impact of a patient’s death extends across the lifetime of the patient and continues onward for loved ones who are still alive, exacting a significant mental, emotional, and physical toll on the family members ^17^. A physician should be able to recognize this and be intentional with how each family needs things explained to them. Often a family needs support in the psychosocial adjustments that take place when a family member is sick. Family caregivers may also experience mental impacts as they support the person who is ill ^18^. Several students talked about how their medical education is very science-based, and that sometimes humanity can be lost during the treatment of their patients as clinicians hyperfocus on treating the diagnosis alone. Students mentioned the importance of understanding the whole patient and, “considering all the factors in their life,” by focusing on biopsychosocial approaches to helping heal the patient. Many appreciated that the class stressed the importance of preserving humanity in medicine.

Respecting patient autonomy and letting patients have a say in their decision-making and end-of-life care was the fifth most common theme mentioned in the papers. Physicians are to prioritize the needs and wants of their patients, but students discussed that it can be challenging to set boundaries. Healthcare providers want to do what is in the best interest of their patients, and sometimes their idea of what is best may not be in line with what the patient wants for themselves. Rather than a hierarchical physician-patient relationship, a collaborative method where both sides share power can lead to an outcome that is best preferred by the sick patient ^19^. At some point, patients may change their decisions about how they want to proceed, and as students recognize that it is a physician’s duty to support and carry out that decision, even if it may conflict with their own professional opinion.

Limitations for this study include a small sample size, as this course has only been active for four semesters, and single institution. There is a potential for selection bias as the students are allowed to rank order their preferences for the humanities courses and those with an existing interest in the subject may positively skew the results. Some students enrolled in the class did not submit their reflection paper and some students did not fill out the one-45 survey on usefulness of the course, thus adding potential confounding. In addition, the endpoints assessed were not clinical but rather surrogate markers were assessed. As such, the impact of such a course on the actual clinical outcomes and the effect on practice of future physicians cannot be ascertained. The course was administered during the preclinical years of medical school, and it is possible that, when presented with actual dying patients, there may be other interventions that could prove more effective in supporting physicians, patients, and their families. Nonetheless, the results from the quantitative and qualitative analyses are encouraging and future studies should address efficacy of such courses in the clinical encounter. 

## Conclusion

The need for education in death, dying, and bereavement in medical education is crucial and students recognize that end-of-life care, death, and dying in general are an extremely important part of becoming a physician. Inadequate or absent training on the effect of death and dying on their patients has the potential to negatively impact their ability to serve as empathic and competent physicians. This transcends specialty and is applicable to almost all physicians in one way or another.

At Cooper Medical School of Rowan University, we have tailored our course to medical students for their success in residency and future career. This course was well liked by participants longitudinally across four semesters. With this, a curriculum that focuses on helping medical students handle difficult conversations and emotions regarding death, dying, and bereavement is essential in medical education.

## Data availability

### Underlying data

OSF: Death and Dying Medical Education Elective Data

https://doi.org/10.17605/OSF.IO/VHBN4 ^8^

This project contains the following underlying data:

- Qualitative Data One45 and Reflections-20220412T133826Z-001.zip
- Reviewing Responses_Themes for REFLECTION PAPERS.xlsx
- stats .docx
- App. B Course Readings Table.pdf

### Extended data

OSF: Death and Dying Medical Education Elective Data

https://doi.org/10.17605/OSF.IO/VHBN4 ^8^

This project contains the following extended data:

- Appendix A.pdf

Data are available under the terms of the Creative Commons Zero "No rights reserved" data waiver (CC0 1.0 Public domain dedication).
