# Supplementary material for: A biopsychosocial approach to death, dying, and bereavement: a course on end-of-life education for medical students
Source: MedEdPublish (2016). 2022 Nov 21;12:44. Originally published 2022 Jun 21. [Version 4] doi: 10.12688/mep.19140.4 (PMC10394390; doi:10.12688/mep.19140.4)
Supplement: Peer review history

# Peer review: Reviewer response for version 4

**DOI:** 10.21956/mep.20794.r33902

**Published:** 2023-08-01

**Authors:** Christy A. Denckla

**Affiliations:** 1 Harvard T.H. Chan School of Public Health, Boston, Massachusetts, USA

Thank you for the opportunity to review this manuscript. Authors report results from a mixed methods study on the experiences and outcomes of students enrolled in a course on end-of-life education.

 This contributes to the medical education community and will interest administrators and educators focused on integrating this very important topic into curriculums. My comments below focus largely on reorganizing the manuscript content and more closely aligning implications and conclusions with the scope of this exploratory study.

**Abstract**

 The first line of the abstract in the background section – perhaps a more strength-based introductory sentence would be useful? Not all students and physicians find end-of-life discussions uncomfortable.

**Introduction**

 The biopsychosocial approach can use some context. The authors do not introduce this framework substantively in the introduction. Doing so will help readers appreciate the conceptual framework that guided this curriculum.

 The introduction could be reorganized to provide background on this study specifically. i.e. this is a mixed methods study on XXX. What is the objective of this study? The authors stop at the background on the necessity for a course (I agree) but then do not give background on the study itself that they go on to describe for the remainder of the paper.

**Results**

 The content on IRB review in the method section should go into the ethics section

 This section could benefit from significant reorganization. One idea is to segment this section into subheadings and organize content around these subheads. For example:

*Study population*

 -This section will describe students, enrollment, sampling, etc.

 -What as the enrollment in each course. Sex %?

*Methods*

 The paragraph that starts with “to understand the impact and response of the course….” This section should have a subheading – this is really the method of the course.

*Measures*

 More information on study measures:

 Survey information

 Reflection papers

 Toronto Empathy Scale

 Budner Tolerance for Ambiguity scale

*Approach to Analysis*

 -Qual analysis

 -quant analysis approach

 -pre/post testing

**Results**

 -The first line repeats content from Methods

 -pre/post testing. Was this analysis done by class (there would be 4 tests in that case) or averaged across all 4 classes? What was the rationale for this approach?

 -Was the comparison range for other “Selective” from Fall 2019-Spring 2021?

**Discussion**

 -Discussion repeats results in many sections. Move content that pertains to results to the results section, and in the discussion, it would be great to see the interpretation of findings and integration with the literature.

 -I would suggest constraining conclusions and generalizability of the study to more closely align with the study methods, which were based on observations made within a small, convenience sample. The extent to which findings generalize to all medical students or other programs is limited, given the study design.

**Limitations section**

 – missing surveys do not necessarily introduce confounding. It does introduce bias.

 -no comparison groups – results are descriptive, with limits to generalizability.

**Conclusion**

 What are future research directions? Follow-up surveys?

Have any limitations of the research been acknowledged?

Partly

Is the work clearly and accurately presented and does it cite the current literature?

Partly

If applicable, is the statistical analysis and its interpretation appropriate?

Partly

Are all the source data underlying the results available to ensure full reproducibility?

Partly

Is the study design appropriate and is the work technically sound?

Partly

Are the conclusions drawn adequately supported by the results?

Partly

Are sufficient details of methods and analysis provided to allow replication by others?

Partly

Reviewer Expertise:

Clinical Psychology and Psychiatric Epidemiology

I confirm that I have read this submission and believe that I have an appropriate level of expertise to confirm that it is of an acceptable scientific standard, however I have significant reservations, as outlined above.

# Peer review: Reviewer response for version 4

**DOI:** 10.21956/mep.20794.r34283

**Published:** 2023-08-01

**Authors:** Rekha Wagani

**Affiliations:** 1 Amity University, Mumbai, Maharashtra, India

I enjoyed reading of the current manuscript and acknowledge the novelty of the study. The study present detailed course contents and its delivery. I can see some of the previous reviewer comments. Some of which were covered however I agree to methodology part which still can be improved. The following points can help author to improve the following manuscript- 

 1. Course type: A six-week humanities course that "covers definitions of death and dying, the process of dying, ethical quandaries, and new concepts of the grieving process." However same is not mentioned across the paper.

 2. English editing is essential in current manuscript.

 3. Research design and qualitative and quantitative data specifications can add more clarity to the manuscript.

 4. The abstract can be rewritten to better express the findings of the current study.

 5. What is the new grieving process in this place? Not clear. As it is not mentioned later in the article.

 6. The argument for course development and instructions required to include relevant citations from the literature in paragraph two. There is one citation for coping, but none for the remaining component like care, hospice etc.

 7. Human Development Dimension which is mentioned in the intro is a little confusing, as in the entire course content (mentioned in the table), no component seems to focus on human development.

 8. The course's goal must be stated consistently throughout. It varies greatly depending on where you go.

 9. The method of data collection and its organization can be mentioned. The IRB approved the project in two sections. Please double-check it. 

 10. If the author used thematic analysis, guidelines can be included here. For example Six steps and coding types, as described by Barun and Clarke (2006), can be mentioned here to provide a better understanding of the data analysis procedure and how the registry has been managed or any other framework utilized by the author.

 11. Death education is clearly mentioned in the discussion section, but it is not mentioned in the introduction. Please make the rationale consistent.

 12. Because the topic and course are extremely sensitive. How the participants' mental health was cared for, as the manuscript claims students found it uncomfortable to discuss the subject. It can be mentioned in procedure.

 13. Dr. Ines Testoni has done a lot of work in this area that is not mentioned anywhere in the discussion. That could be relevant and useful in this case.

Have any limitations of the research been acknowledged?

Yes

Is the work clearly and accurately presented and does it cite the current literature?

Partly

If applicable, is the statistical analysis and its interpretation appropriate?

Partly

Are all the source data underlying the results available to ensure full reproducibility?

Partly

Is the study design appropriate and is the work technically sound?

Partly

Are the conclusions drawn adequately supported by the results?

Partly

Are sufficient details of methods and analysis provided to allow replication by others?

Partly

Reviewer Expertise:

Mixed method, both qual and quant, death education, suicide prevention, intervention studies, sensing and mental health

I confirm that I have read this submission and believe that I have an appropriate level of expertise to confirm that it is of an acceptable scientific standard, however I have significant reservations, as outlined above.

# Peer review: Reviewer response for version 4

**DOI:** 10.21956/mep.20794.r34289

**Published:** 2023-07-26

**Authors:** Paul Victor Patinadan

**Affiliations:** 1 Nanyang Technological University, Singapore, Singapore

The manuscript considers a 6-week ‘selective’ course focusing on the topics of death, dying and bereavement for medical students. Qualitative analyses were employed with participant reflections as the data points. Quantitative data was also collected and analysed. The authors were clear in describing in detail the content and instruction for their Selective.

 The manuscript in its current form has already seen some revision, and I believe it is very close to reaching a successful publication standard. I thank the previous reviewers for their comprehensive work, and looking through their comments, am in general agreement with their views. The current authors’ work is an important undertaking, as dealing with EoL issues will be always be a vocational prerogative for healthcare professionals. The merits of the piece are its succinct literature review, detailed curricula example, and comprehensive discussion.

 I do, however, feel that the qualitative methods and analysis require a little more detail. Was there an ontological/epistemological stance employed when dealing with the data (constructivist etc.)? How were a priori themes generated and agreed upon? What software was employed in this process? Some minor inclusions to showcase the rigour of the work.

 Additionally, the authors mention that quantitative analyses were carried out. I feel that without any statistical analyses and interpretation, one cannot deem descriptive data (such as whether students found the course useful) as truly quantitative. There were pre-post test analyses involved (Empathy/Ambiguity scales), but these were only very briefly mentioned as not significant and not discussed again. Non-significant findings are also useful and can provide critical insight, especially if they might be at odds with the qualitative data analyzed. In this case, these numbers can show that proceeding through a structured curriculum isn’t enough to impact the deep-set values of empathy or practical wisdom of dealing with ambiguity. These might come later from practice and immersion in the field (similar to what another reviewer had mentioned). These are some insights that can be considered in the discussion.

 Overall, this is a very useful manuscript for the journal’s audience, just a little more critical insight would be adequate to push the piece to successful publication.

Have any limitations of the research been acknowledged?

Yes

Is the work clearly and accurately presented and does it cite the current literature?

Yes

If applicable, is the statistical analysis and its interpretation appropriate?

Partly

Are all the source data underlying the results available to ensure full reproducibility?

Yes

Is the study design appropriate and is the work technically sound?

Yes

Are the conclusions drawn adequately supported by the results?

Yes

Are sufficient details of methods and analysis provided to allow replication by others?

Partly

Reviewer Expertise:

Mixed-methods research, Thanatology and End of Life issues, Health Professions Education, Pedagogy Development, Medical Humanities

I confirm that I have read this submission and believe that I have an appropriate level of expertise to confirm that it is of an acceptable scientific standard, however I have significant reservations, as outlined above.

# Peer review: Reviewer response for version 3

**DOI:** 10.21956/mep.20671.r32507

**Published:** 2022-09-20

**Authors:** Kelly Melekis

**Affiliations:** 1 Social Work, Skidmore College, Saratoga Springs, NY, USA

Overall, the authors contribute to the body of literature regarding medical education on death, dying, and end-of-life care.  As the authors note, there is still a relative dearth of courses/curricula on this topic despite its universal nature. 

 My main concerns about this manuscript are as follows:

1. There is insufficient detail on the methods, particularly the qualitative methods used to analyze the reflection papers.  The authors discuss themes, however, there is not enough detail to assess how these themes were determined (or how they were determined a priori) and whether the method was sound.  Related, I would recommend edits to the tables, particularly Tables 2 and 3.  Table 2 includes a sample of comments on the course, however, a table with sample quotes that represent specific themes would be more useful. Table 3 is a quantification of the qualitative responses.  First, this reads as a list of topics or words, not themes.  Again, this speaks to a lack of methodological clarity.  I would recommend further analysis of the qualitative data following clarification of the qualitative method utilized. 
2. While the authors raise some important limitations, I think it is important to acknowledge the potential impact of the context of COVID on the data collected. Given that the majority of data (but not all) was collected during COVID, it seems worth noting that this could have an impact on how medical students approach and evaluate a selective on death and dying. 

Have any limitations of the research been acknowledged?

Yes

Is the work clearly and accurately presented and does it cite the current literature?

Yes

If applicable, is the statistical analysis and its interpretation appropriate?

Yes

Are all the source data underlying the results available to ensure full reproducibility?

Yes

Is the study design appropriate and is the work technically sound?

Partly

Are the conclusions drawn adequately supported by the results?

Partly

Are sufficient details of methods and analysis provided to allow replication by others?

No

Reviewer Expertise:

gerontology, end-of-life care, end-of-life education, health and well-being of vulnerable older adults, health impacts of housing and social environment, interprofessional practice, evaluation, qualitative methods

I confirm that I have read this submission and believe that I have an appropriate level of expertise to state that I do not consider it to be of an acceptable scientific standard, for reasons outlined above.

# Response

**Published:** 2022-11-04

**Authors:** Simran Kripalani

**Affiliations:** Cooper Medical School of Rowan University, USA

*Overall, the authors contribute to the body of literature regarding medical education on death, dying, and end-of-life care. As the authors note, there is still a relative dearth of courses/curricula on this topic despite its universal nature.*

 Thank you. We appreciate the reviewers assessment of the topic and its appropriateness in medical education.

*My main concerns about this manuscript are as follows: There is insufficient detail on the methods, particularly the qualitative methods used to analyze the reflection papers.*

*The authors discuss themes, however, there is not enough detail to assess how these themes were determined (or how they were determined a priori) and whether the method was sound.*

 Thank you. We added greater clarity to the Methods section and hope this addresses the reviewer’s concerns.

*Related, I would recommend edits to the tables, particularly Tables 2 and 3. Table 2 includes a sample of comments on the course, however, a table with sample quotes that represent specific themes would be more useful. Table 3 is a quantification of the qualitative responses. First, this reads as a list of topics or words, not themes. Again, this speaks to a lack of methodological clarity.*

*I would recommend further analysis of the qualitative data following clarification of the qualitative method utilized.*

 We thought it best to eliminate Table 2, based on the reviewer’s comments. We also restructured the second table and organized the thematic content under “provider-centric” and “patient-centric” comments.

*While the authors raise some important limitations, I think it is important to acknowledge the potential impact of the context of COVID on the data collected. Given that the majority of data (but not all) was collected during COVID, it seems worth noting that this could have an impact on how medical students approach and evaluate a selective on death and dying.*

 Thank you and we agree that COVID-19 has had significant, perhaps as yet uncharacterized impacts on our students and residents. We added this to our “Limitation” section. We hope that these revisions are acceptable but would be happy to further adapt the manuscript at your discretion.

# Peer review: Reviewer response for version 3

**DOI:** 10.21956/mep.20671.r32489

**Published:** 2022-09-01

**Authors:** Anastasia Kunac, Sheila Rugnao

**Affiliations:** 1 Division of Trauma and Surgical Critical Care, Department of Surgery, Rutgers New Jersey Medical School, Newark, NJ, USA; 2 Rutgers New Jersey Medical School, Newark, NJ, USA

We have no further comments/concerns related to this publication.

Have any limitations of the research been acknowledged?

Yes

Is the work clearly and accurately presented and does it cite the current literature?

Partly

If applicable, is the statistical analysis and its interpretation appropriate?

Yes

Are all the source data underlying the results available to ensure full reproducibility?

No source data required

Is the study design appropriate and is the work technically sound?

Partly

Are the conclusions drawn adequately supported by the results?

Yes

Are sufficient details of methods and analysis provided to allow replication by others?

Yes

Reviewer Expertise:

Education, Palliative Care, General Surgery, Trauma, Surgical Infection

We confirm that we have read this submission and believe that we have an appropriate level of expertise to confirm that it is of an acceptable scientific standard.

# Peer review: Reviewer response for version 2

**DOI:** 10.21956/mep.20621.r32387

**Published:** 2022-08-10

**Authors:** Anastasia Kunac, Sheila Rugnao

**Affiliations:** 1 Division of Trauma and Surgical Critical Care, Department of Surgery, Rutgers New Jersey Medical School, Newark, NJ, USA; 2 Rutgers New Jersey Medical School, Newark, NJ, USA

We have reviewed the recent revisions.  There are a few minor issues that require attention.  We would recommend conditional approval provided that these discrepancies can be addressed:

 The abstract should be consistent, numerically, with the results and methods section. (ie the N # of people included in the various assessments) 

 The discussion section has been edited for readability, however, there is still a discrepancy on the N number of people included for the Likert survey and the reflective paper. The first paragraph states that only 3 semesters were used for the Likert survey, but that in the survey 49/54 agreed the course was useful. The second paragraph of the results section states there were 42 respondents for the survey and 41 for the reflection paper. However, there is a total N of 54 for all semesters and a total number N=54 included in Table 3 (qualitative responses for reflection papers).

Have any limitations of the research been acknowledged?

Yes

Is the work clearly and accurately presented and does it cite the current literature?

Partly

If applicable, is the statistical analysis and its interpretation appropriate?

Yes

Are all the source data underlying the results available to ensure full reproducibility?

No source data required

Is the study design appropriate and is the work technically sound?

Partly

Are the conclusions drawn adequately supported by the results?

Yes

Are sufficient details of methods and analysis provided to allow replication by others?

Yes

Reviewer Expertise:

Education, Palliative Care, General Surgery, Trauma, Surgical Infection

We confirm that we have read this submission and believe that we have an appropriate level of expertise to confirm that it is of an acceptable scientific standard, however we have significant reservations, as outlined above.

# Peer review: Reviewer response for version 1

**DOI:** 10.21956/mep.20498.r32259

**Published:** 2022-07-18

**Authors:** Anastasia Kunac, Sheila Rugnao

**Affiliations:** 1 Division of Trauma and Surgical Critical Care, Department of Surgery, Rutgers New Jersey Medical School, Newark, NJ, USA; 2 Rutgers New Jersey Medical School, Newark, NJ, USA

The work by Dr. Kripilani and colleagues is novel, important, and should be part of any medical school curriculum.  The authors are to be commended on their commitment to provide comprehensive education around death and dying as it is the natural consequence of living and not explored frequently or adequately by those who practice medicine.

 We have several concerns about the article in its current form.  The manuscript appropriately cites current literature, but some of the language is not clear. There are a number of grammatical errors, and incomplete sentences that should be addressed, including a misspelling in the title of figure 1.

 A mixed methods approach to evaluating the course is presented.  This approach is reasonable, but it is difficult to parse out the results of the study as they are presented in part within the Methods section and only sparsely in the Results section.  It would be helpful to illustrate how the representative student comments were sifted/sorted to the themes identified.  Further, the Methods section should include a reference to IRB approval for the study which would imply that the medical students gave consent for their coursework to be used in this manner.

 The methods section also describes the Likert scale that was used to evaluate the usefulness of the course. The paper states the information is shown in Table 3, however, table 3 is a qualitative evaluation on themes that were presented in the reflection papers.  

 Table 2 is described in the text as “themes that were most prominent” however the table includes selected specific verbatim comments, not themes.

 Much of the information presented in the discussion should be included in the results section in a clear and concise manner. There is a discrepancy of the total number of participants, responders, and number included in Table 3. These discrepancies should be addressed ((N=50 in table 3, but 57 participants and 54 responders noted elsewhere).

 The limitations of the study are clearly articulated and the conclusions drawn are mostly reasonable.  The authors make a jump in the final sentence of the paper that learning about death and dying will translate to the students' ability to have difficult conversations about death and dying.  This is NOT a reasonable conclusion unless those skills are explicitly taught and evaluated.  Simply learning the subject matter does not necessarily translate into good physician-patient or physician-family communication.  Simulated encounters could be used to ascertain if the students are able to "put it all together" and translate their knowledge to clinical skills, but that seems beyond the scope of the curriculum presented.

Have any limitations of the research been acknowledged?

Yes

Is the work clearly and accurately presented and does it cite the current literature?

Partly

If applicable, is the statistical analysis and its interpretation appropriate?

Yes

Are all the source data underlying the results available to ensure full reproducibility?

No source data required

Is the study design appropriate and is the work technically sound?

Partly

Are the conclusions drawn adequately supported by the results?

Yes

Are sufficient details of methods and analysis provided to allow replication by others?

Yes

Reviewer Expertise:

Education, Palliative Care, General Surgery, Trauma, Surgical Infection

We confirm that we have read this submission and believe that we have an appropriate level of expertise to state that we do not consider it to be of an acceptable scientific standard, for reasons outlined above.
